# Haplotype Block Structure Is Conserved across Mammals

**DOI:** 10.1371/journal.pgen.0020121

**Published:** 2006-07-28

**Authors:** Victor Guryev, Bart M. G Smits, Jose van de Belt, Mark Verheul, Norbert Hubner, Edwin Cuppen

**Affiliations:** 1 Hubrecht Laboratory, Utrecht, The Netherlands; 2 Max-Delbruck-Center for Molecular Medicine (MDC), Berlin-Buch, Germany; University of Oxford, United Kingdom

## Abstract

Genetic variation in genomes is organized in haplotype blocks, and species-specific block structure is defined by differential contribution of population history effects in combination with mutation and recombination events. Haplotype maps characterize the common patterns of linkage disequilibrium in populations and have important applications in the design and interpretation of genetic experiments. Although evolutionary processes are known to drive the selection of individual polymorphisms, their effect on haplotype block structure dynamics has not been shown. Here, we present a high-resolution haplotype map for a 5-megabase genomic region in the rat and compare it with the orthologous human and mouse segments. Although the size and fine structure of haplotype blocks are species dependent, there is a significant interspecies overlap in structure and a tendency for blocks to encompass complete genes. Extending these findings to the complete human genome using haplotype map phase I data reveals that linkage disequilibrium values are significantly higher for equally spaced positions in genic regions, including promoters, as compared to intergenic regions, indicating that a selective mechanism exists to maintain combinations of alleles within potentially interacting coding and regulatory regions. Although this characteristic may complicate the identification of causal polymorphisms underlying phenotypic traits, conservation of haplotype structure may be employed for the identification and characterization of functionally important genomic regions.

## Synopsis

Differences at the DNA level are the major contributant underlying the phenotypic diversity between individuals in a population. The most common type of this genetic variation are single nucleotide polymorphisms (SNPs). Although the majority of SNPs do not have a functional effect, others may affect chromosome organization, gene expression, or protein function. SNPs and their individual states (alleles) are not randomly distributed throughout the genome and within a population. Recombination and mutation events, in combination with selection processes and population history, have resulted in common block-like structures in genomes. These structures are characterized by a common combination of SNP alleles, a so-called haplotype. Selection for specific haplotypes within a population is primarily driven by the advantageous effect of an individual polymorphism in the haplotype block.

By comparing the orthologous rat, mouse, and human haplotype structure of a 5-megabase region from rat Chromosome 1, the authors now show that haplotype block structure is conserved across mammals, most prominently in genic regions, suggesting the existence of an evolutionary selection process that drives the conservation of long-range allele combinations. Indeed, genome-wide gene-centric analysis of human HapMap data revealed that equally spaced polymorphic positions in genic regions and their upstream regulatory regions are genetically more tightly linked than in non-genic regions.

These findings may complicate the identification of causal polymorphisms underlying phenotypic traits, because in regions where haplotype structure is conserved, not a single polymorphism, but rather combinations of tightly linked polymorphisms could contribute to the phenotypic difference. On the other hand, conservation of haplotype structure may be employed for the identification and characterization of functionally important genomic regions.

## Introduction

Haplotype maps describe common patterns of genetic variation of genomes and have important applications in the design and analysis of genetic experiments [1–3], such as disease-susceptibility mapping efforts. The three major processes that shape haplotype structure are mutation, recombination, and selection. Together with population history, they establish the great distinction of haplotype patterns observed in mammalian genomes [4–6]. Recombination events define the borders of the linkage disequilibrium (LD) blocks. This is supported by a strong correlation between LD breakpoints and recombination hotspots [7,8]. On the other hand, population history largely determines the size of the blocks. Humans, with a relatively heterogeneous founder population, have small blocks with a median size of 45 kilobases (kb), and inbred populations of laboratory mice, which experienced a recent genetic bottleneck during domestication, have large blocks spanning hundreds of kilobases.

The role of selection in shaping the haplotype block organization is not clearly understood, given the relatively small number of loci with strong proof for being under selection pressure. A recent search for LD landscapes that exhibit signs of positive selection identified as many as 1,800 genes in the human genome [9]. On the other hand, a similar selection case reported previously [10] is considered equally consistent with neutral evolution by other investigators, because LD patterns in this region do not stand out as exceptional relative to other loci across the genome [11].

Comparative genomics may provide a powerful approach to study the role of selection in shaping genomic segments with limited haplotype diversity. For the human, a detailed genome-wide haplotype map is already available [5]. Similar programs have been initiated for the mouse and the rat [3] (N. Hubner, personal communication), but no genome-wide high-resolution genotyping data are currently available for all three species. Therefore, we have chosen to study a 5-megabase (Mb) genomic region in which the LD structure has been characterized in detail in relation to an anxiety quantitative trait locus (QTL) in mouse [12]. Here, we present the haplotype structure for the orthologous rat genomic segment, and show that there is significant overlap in block structure between rat, mouse, and human, which suggests a selective mechanism may be driving haplotype block organization in mammals.

## Results/Discussion

**Figure 1:**
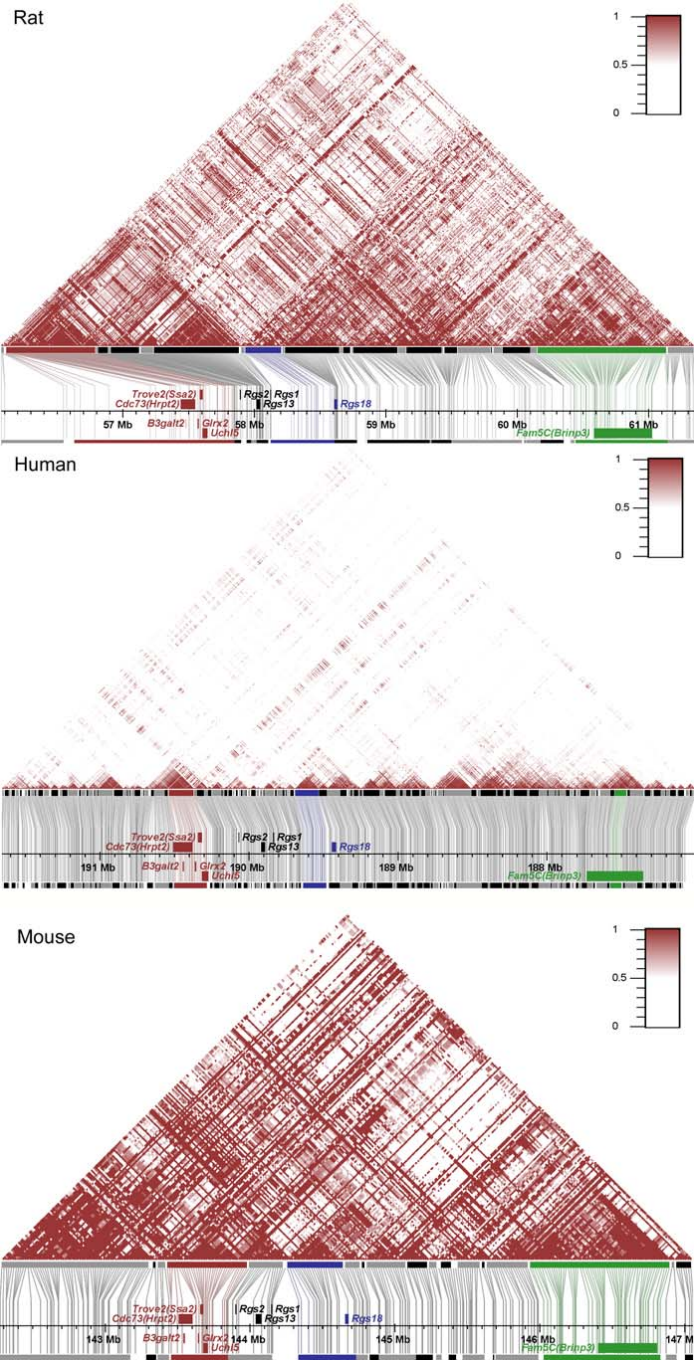
Patterns of LD for Orthologous Genomic Segments of Approximately 5 Mb in Rat, Human, and Mouse LD plots for orthologous genomic segments in rat (A), human (B), and mouse (C) are shown. For each panel, the following information is shown: LD plot (top), haplotype blocks in SNP coordinates (middle), and physical map and haplotype blocks in physical coordinates (bottom). The haplotype map has a gradient representation for |D′| values that assists visual comparison of haplotype structure. Haplotype blocks were built with stringent criteria, sometimes resulting in splitting of visually recognized blocks. Three characteristic haplotype blocks that are conserved cross-species have been color-coded and are discussed in the text. Similar plots for a second mouse set, two other human populations, and the combined human set are available as Figures S1 and S2.

The 5-Mb genomic region that is compared in this study is located on mouse Chromosome 1 and has uninterrupted synteny to rat Chromosome 13 and human Chromosome 1 (Table 1). We resequenced a total of about 300 kb dispersed through this region in 41 laboratory rat strains (total sequence length of 12.3 Mb) and discovered 1,351 single nucleotide polymorphisms (SNPs). This information was used to build an LD and haplotype map of this region for the rat (Figure 1A). HapMap Phase I data were used to generate a similar map for the combined human populations (Figure 1B; independent maps for the European (CEU), African (YRI), and Asian (CHB+JPT) populations are provided in Figure S1). For the mouse map, additional data to that obtained by Yalcin and coworkers [12] became available and were included in the present analysis encompassing preliminary genotyping data (48 mouse inbred strains) obtained as part of the mouse HapMap project [3] (Figure 1C). Our analysis of this mouse dataset resulted in a similar block structure compared to that obtained originally by Yalcin and coworkers (Figure S2).

**Table 1:**
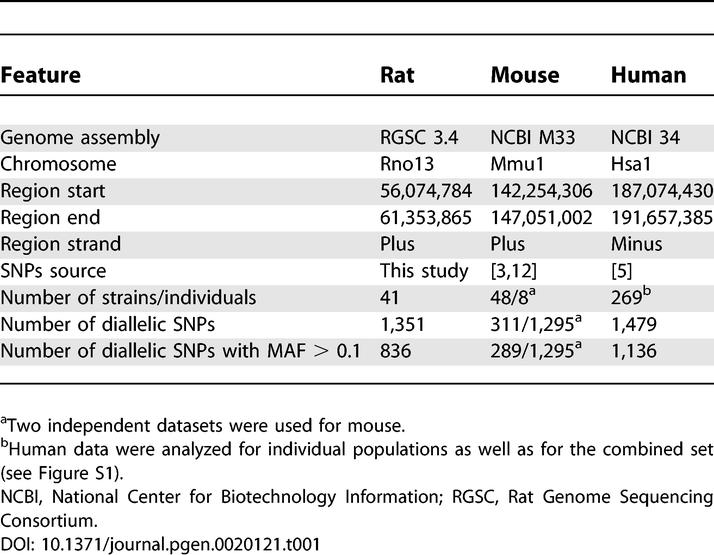
Data Used in Haplotype Block Analysis

### Conserved Haplotype Structure in Mammals

Overall, LD spans larger segments in the mouse and rat, compared to the human. Larger haplotype blocks reflect the fundamental differences in population history between human and inbred laboratory animals. Strikingly, LD patterns in the rat and mouse inbred strains have common features. Both organisms exhibit extended blocks of increased LD corresponding to the following genomic segments: (1) the cluster of five genes: *B3galt2, Cdc73 (Hrpt2), Glrx2, Trove2 (Ssa2),* and *Uchl5;* (2) the large *Fam5C (Brinp3)* gene; and (3) regions flanking the *Rgs18* gene.

**Figure 2:**
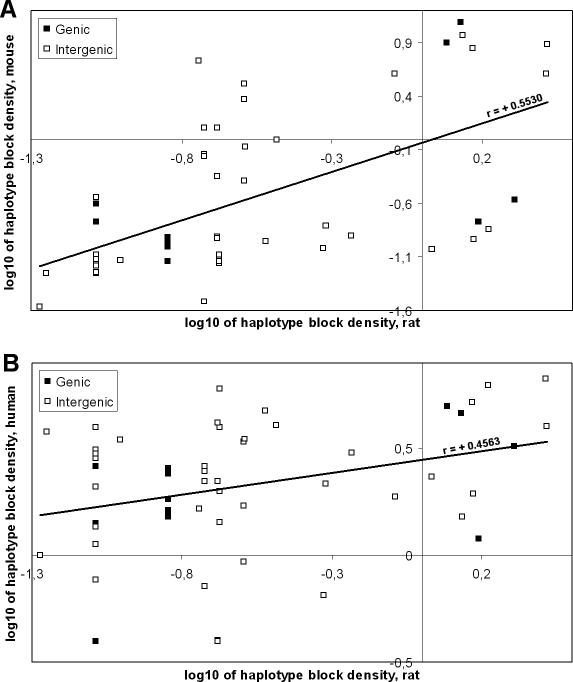
Comparison of the Haplotype Block Densities between Syntenic Regions of Rat, Mouse, and Human (Same Genome Segments as Shown in Figure 1) The scatter plots show log_10_ of the amount of haplotype blocks per 100-kb bin in rat (horizontal) against log_10_ of the amount of haplotype blocks seen in syntenic region of mouse (A) and human (B) genome (vertical). Data points for gene-containing and intergenic genomic bins are shown as closed and open blocks, respectively. Observed correlations of haplotype block densities are significant in linear (*r* = +0.5530; *p* < 0.0001 [A] and *r* = +0.4563; *p* = 0.0005 [B]) as well as in log-transformed space (*r* = +0.6795; *p* < 0.0001 [A] and *r* = +0.3209; *p* = 0.0180 [B]).

Although the human haplotype structure is characterized by much smaller blocks, the most extensive human regions displaying high LD, and thus extended haplotype blocks, include the cluster of five genes mentioned above, the coding part of *Fam5C (Brinp3),* and the region flanking *Rgs18* (Figure 1B). Since rat, mouse, and human genomes rarely share the same polymorphic positions, direct comparison of their LD values is not possible. We applied a sliding-window approach [13] to analyze the overlap in block structure in pairwise comparisons. We found that there are significant correlations (*p* < 0.05) between haplotype block partitioning for the rat, human, and mouse (Table 2). The interspecific comparison of haplotype structure shows correlation of haplotype block density between syntenic regions regardless of gene content (Figure 2). Interestingly enough, we observe many intergenic regions that consistently exhibit strong LD in two species. For all three species, there is a negative association, although not significant, between haplotype block density and the number of genic bases (Pearson correlation *r* = −0.17, *p* = 0.21; *r* = −0.11, *p* = 0.43; and *r* = −0.22, *p* = 0.10 for rat, mouse, and human, respectively).

**Table 2:**
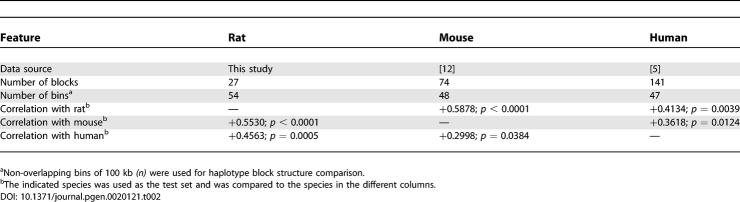
Characteristics and Correlation of Haplotype Structures between Datasets

### Selection Governs the Conservation of Haplotype Blocks

Interestingly, conservation of haplotype structure suggests that the degree of LD is consistent between syntenic segments. Three different mechanisms could explain the observed similarity in haplotype block organization and the tendency of genic regions to reside in high LD segments. First, the fine-scale conservation of recombination rate would result in similar haplotype block structures in different organisms. Although recombination hotspots may very well explain the similarity in LD structure for different human subpopulations [14,15], recombination hotspots have been shown to evolve rapidly [16] and have been found not to be conserved very strongly, even between closely related organisms such as the human and chimpanzee [17,18]. In addition, the presence of a recombination hotspot alone is not sufficient for splitting haplotype blocks. Although boundaries of haplotype blocks correlate strongly with recombination hotspots [7,8], direct sperm typing and indirect coalescent analyses show that haplotypes do not break at every recombination hotspot [5,16].

Secondly, suppression of recombination in specific genomic segments, such as gene coding regions, would also result in the observed conservation pattern. The only way to study recombination as an isolated process is by direct sperm typing to characterize individual meiotic crossover events [7,16,19]. We have analyzed the relationship between crossover positions and gene features using the most extensive dataset currently available [8]. We found that the regions where recombination events were observed are enriched in gene sequences (e.g., 47.7% of these segments are genic as compared to 39.4% for the complete 2.5-Mb segment that was studied), instead of depleted, arguing against a major contribution of this mechanism. This observation is in a good agreement with the previous finding that recombination rate positively correlates with gene features [19].

As a remaining mechanism, similar selection processes acting on large genomic segments in different mammalian species could result in resemblance of haplotype structure. However, the genomic region investigated in the present study does not exhibit evident signatures of selection in human populations [20,21], nor in human and chimp lineages [22], suggesting that subtle selection such as selective sweeps, background selection, or haplotype-driven selection (i.e., selection for combinations of specific alleles at different genomic positions) may largely determine the observed extended haplotype block length in genic regions. Previous analysis of LD patterns in three chimpanzee regions identified only limited overlap in the locations of LD breakdown between chimpanzee and human [17]. However, this study was based on rather small genomic regions of about 500 kb and did not allow for efficient comparisons between LD in genic and intergenic segments.

### Gene-Centered Analysis of LD Decay Reveals Signatures of Haplotype-Driven Selection

It is intriguing to speculate that selection drives the conservation of haplotype structure in mammalian genomes. Evidently, selection would have the highest impact on haplotype structure in functionally important regions of the genome. However, it is unclear if selective sweeps and background selection alone can cause this kind of conservation or whether haplotype-driven balancing selection is required to ensure long-term preservation of haplotype structure. The current human haplotype map is sufficiently dense and complete, thereby allowing for the genome-wide inspection for signals indicative of selection processes. The initial, LD-centered analysis of HapMap data suggested that regions with high and low LD are enriched in genic sequences [5]**.** We reanalyzed the HapMap phase I data using a gene-centered approach to investigate if extended LD in functionally important regions is a general property in the human genome and whether long-range profile of LD decay has an asymmetrical distribution in 5′- and 3′-flanks of a gene, a possible indication of haplotype-driven selection.

**Figure 3:**
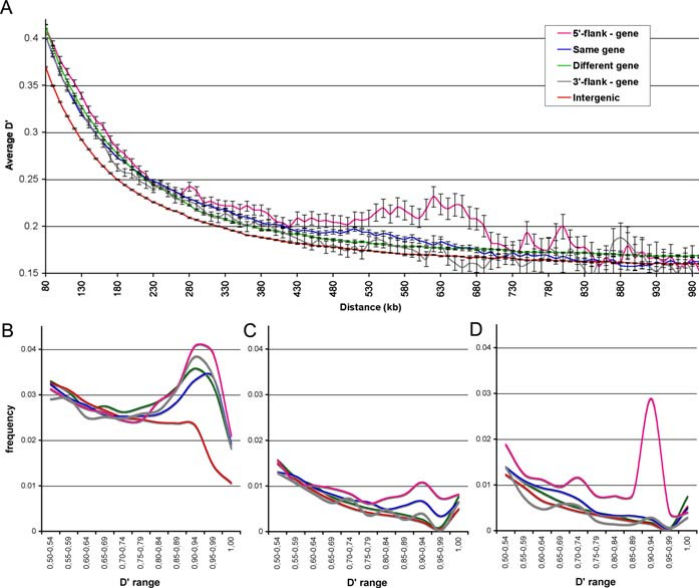
Analysis of LD Decay for Functionally Different Segments of the Human Genome (A) The graph shows average values of |D′| and their confidence limits (± standard deviation) as a function of the physical distance between SNPs for the following categories: (1) both SNPs reside in the same gene (blue line), (2) the SNPs reside in two different genes (green line), (3) both SNPs reside in the same intergenic region (red line), (4) one SNP resides in the gene and the other in the 30 kb upstream region of the same gene (purple line), and (5) one SNP resides in the gene and the other in the 30 kb downstream region of the same gene (gray line). (B) Frequency distribution spectrum of |D′| values for SNP pairs at 100-kb distance. High |D′| values (>0.8) are overrepresented for equally spaced SNPs in a gene and its flanking regions as compared to intergenic regions. (C) Frequency distribution of high LD values (|D′| > 0.5) for SNP pairs at 450-kb distance. Higher LD values are observed between a gene and its upstream region. (D) Frequency distribution of high LD values (|D′| > 0.5) for SNP pairs at 650-kb distance. Higher LD values are observed between a gene and its upstream region. The bin with |D′| = 1 is isolated to a separate bin in panels (B–D) as there is a considerable frequency bias for this |D′| value. Similar graphs plotted for separate human populations are available as Figures S3, S4, and S5.

Comparison of LD decay profiles for different functional parts of the human genome revealed slower LD decay in genic segments and their flanking regions than in intergenic segments (Figure 3A). The tendency of reduced recombination rate, or stronger LD within and close to genes, was noted in previous studies [13,23,24], but was not documented in detail. Our analysis shows that the LD decay profiles substantially vary among different genomic partitions. Strikingly, the higher LD values for genic regions are attributed to a prominent component of very high LD values (|D′| > 0.8; Figure 3B), rather than to a generally elevated LD over the complete spectrum. The presence of this component in different data partitions evolves with distance between polymorphic loci. Over a short distance (e.g., 100 kb; Figure 3B), all gene-related but not intergenic regions are characterized by overrepresentation of very high LD values, potentially reflecting the consequences of selective sweeps or background selection. With increasing distance between polymorphic positions over 200 kb, we observe the highest LD values for SNPs residing in genic regions and their upstream loci, whereas the linkage with the downstream region gradually falls to levels characteristic of intergenic segments of the genome (Figure 3C and 3D). Furthermore, elevated LD over long distances was also found for loci located within the same gene (Figure 3C). In both cases, a pronounced component of high LD values is underlying the observed increase.

The observed asymmetry of LD towards promoter regions of genes and increased LD within individual genes further supports the influence of haplotype-driven selection on organization of variation in mammalian genomes. This mechanism could drive the elimination of recombinants between specific alleles from a population, resulting in limited haplotype diversity and extended blocks encompassing segments on which the selection acts. Genetic variants that individually act as weak modulators of phenotypes, but exhibit more profound biological effects in specific allele combinations, could form the basis for haplotype-driven selection. These presumptive combinations can include synergistic compensatory alleles that underlie co-evolution of interacting residues in mammalian proteins [25], as well as compensatory alleles that up- or down-regulate the expression of genes with hypo- or hyper-morphic alleles, respectively. An illustrative example that conforms to the idea of selection for haplotypes (i.e., conservation of specific allele combinations) is the split of QTL into several sub-QTLs when extremely high-resolution genotyping was applied [1].

### Excess of SNPs Located at Syntenic Positions in Mouse and Rat

Whereas signatures of selective sweeps exhibit reduced divergence at the nucleotide level, haplotype-driven selection is expected to result in an excess of polymorphisms located at syntenic positions. To test this assumption, we compared positions of polymorphisms using rat–mouse alignment. Based on the observed polymorphism frequency of one SNP per 223 base pairs (bp) in the resequenced segments of rat Chromosome 1 and the occurrence of 923 SNPs in the orthologous mouse segments, one would expect only 4.1 SNPs at exactly the same nucleotide positions in mouse and rat when assuming a random SNP distribution. However, inspection of our experimental dataset revealed nine SNPs at syntenic positions in both species, which is significantly more than expected (*p* = 0.0252). Interestingly, five of the “conserved” SNPs retain the same nucleotide variants in mouse and rat (Dataset S1). Furthermore, of those five, two are located in extended and conserved haplotype block encompassing five genes, and one is in an intron of *Fam5C* gene that also exhibits strong LD in all three species. The observed excess of SNPs at syntenic positions in rat and mouse provides an additional evidence of haplotype-driven selection. It implies that many more polymorphisms descendent from the murine common ancestor, including functionally important variants, are yet to be found upon the arrival of rat and mouse genome-wide SNP data.

### Implications for Genetic Association Studies

The observed haplotype block conservation has several implications for experimental approaches. First, it may complicate genetic studies, as identification of a single causal polymorphism underlying a QTL may turn out to be unfeasible, and focus may switch to combinations of tightly linked alleles. Secondly, multi-specific approaches, using several model organisms at a time for narrowing down the QTLs region, may be less effective than anticipated.

On the other hand, maps of conserved haplotype structures could point towards genomic segments that are under clear selection pressure in mammalian species and may allow for the identification of functional genomic elements, including important promoter and enhancer regions. From this perspective, the ongoing efforts for constructing mouse [3] and rat (N. Hubner, personal communication) haplotype maps will not only provide valuable tools for genetic mapping and association experiments, but will also result in a resource that can be used to gain more insight into the organization of mammalian genomes. Functionally important genomic regions could be revealed using a systematic interspecies comparison of haplotype structure, in combination with sequence alignments and genome annotation.

## Materials and Methods

### Sample sources, DNA isolation, and sequencing.

The specimens of rat laboratory strains were obtained from commercial breeders. We have used 41 isolates of commonly used strains: *AO/OlaHsd; AUG/OlaHsd; BBwortky; BDE/Ztm; BDII/Ztm; BDIV; BDIX/Ztm; BH/Ztm; BS/Ztm; CDR/Y; DON; DRH/Seac; F344; GAERS; GRslc; HAA; HTX/Kyo; HWYslc; IS/Kyo; KDP; LAA; LEA; LEC; LEW/Ztm; LUDW/OlaHsd; MES; MHS; NER; RCS/Kyo; RICO/Ngs; SER; SHHF; THE; TRM; WAG/RijHsd; WBN/KobSlc; WF; WIAR; WKA/Seac; WK;* and *WNA.*

DNA isolation was performed using phenol-chloroform extraction, followed by isopropanol precipitation, as previously published [26]. For the mouse anxiety QTL region located on Chromosome 1 and the syntenic regions of the rat and human genomes studied (Table 1), we constructed a three-way alignment using Multi-LAGAN [27]. By mapping mouse SNP-harboring regions [12] to rat genomic sequence, we designed 384 amplicons of approximately 1,000 bp each. We amplified and sequenced these amplicons in 41 rat inbred laboratory strains. In total, approximately 300 kb of sequence was generated, resulting in the identification of 1,351 SNPs. At least 85% of the genotypes were obtained for these SNPs for each of the strains.

### SNP discovery and genotyping.

We performed SNP discovery by comparing sequencing reads against Rat Genome Sequencing Consortium (RGSC) 3.4 rat genome assembly (http://www.hgsc.bcm.tmc.edu/projects/rat). Polymorphic positions with Phred qualities of 20 and more were automatically genotyped in every strain. The automatic genotyping was manually verified by visual inspection of chromatogram data. In total, we genotyped 1,351 SNPs by sequencing of approximately 300 kb of genomic sequence. We performed two rounds of resequencing for samples with ambiguous or low-quality sequence calls. As a result, we were able to obtain at least 85% of genotypes for each strain.

### Datasets used in the study.

Genotyping data for the rat laboratory strains (1,351 SNPs genotyped in 41 inbred strains) was generated as a part of this study. For mouse, we used publicly available datasets (1) described in [12] (1,295 SNPs genotyped in 8 inbred strains) and (2) resource introduced in [3] (311 SNPs genotyped in 48 inbred strains) and human HapMap public release 19 [5] (complete set of 1,479 SNPs genotyped in 269 individuals that was also analyzed as three subsets of European, CEU, 90 individuals; African, YRI, 90 individuals; and Asian, CHB+JPT, 89 individuals origin).

### The choice of statistic for LD measures.

For our analysis, we have focused on recombination rather than mutation history. Therefore, we chose a commonly used standardized gametic disequilibrium coefficient |D′| as a measurement for LD. This measurement is not significantly affected by allele frequencies [28], although it is known to fluctuate upward when the number of samples is small. Thus, being too conservative, it did not provide enough resolution for the mouse data presented in [12], which contains genotypes for only eight strains (of which two had the same haplotype throughout the entire region studied). On the other hand, the mosaic nature of the mouse genome results in an extremely small variation rate of about 0.5 SNP per 10 kb at segments inherited from same subspecies [4]. Thus, we conclude that contribution of mutations in mouse haplotype structure is far less than that of recombination, consequently *r* ^2^ rather than a |D′| measure would provide a more sensitive and comprehensive view on the recombination history of eight inbred mouse strains. For the second set of mouse data [3], genotyping data for 48 strains is available, and although the SNP density is less, the use of |D′| is appropriate. Not surprisingly, a visual comparison between the dataset from [12] with *r^2^* statistics and the data from [3] confirms the similar haplotype structure of the investigated region.

### Haplotype block construction**.**

We used only common SNPs with minor allele frequencies of at least 0.1. Haplotype block partitioning was based on evidence for historical recombination [29]. We calculated confidence intervals for LD measures using 1,000 bootstrap iterations. There is strong evidence for historical recombination between markers if their upper 95% confidence bound of LD measure is less than 0.9. Further, we defined a haplotype block as a region over which a very small proportion (<5%) of comparisons among informative SNP pairs show strong evidence for historical recombination. We considered all possible blocks of physically consecutive SNPs through the region and ranked them by the number of SNPs included. We started with blocks containing a maximal number of SNPs and excluded all blocks that physically overlapped with it. This process was repeated until we selected the set of non-overlapping blocks.

### Interspecific comparison of haplotype structure.

For pairwise haplotype structure comparison, we used a sliding-window approach. We defined the haplotype blocks as described above and performed interspecific comparisons of haplotype block density between syntenic segments. We calculated the number of defined haplotype blocks for every non-overlapping 100-kb window (54 windows total) of the rat genome segment (reference set). Using three-way alignment of rat, mouse, and human genome segments, we then projected every 100-kb window of the reference set on genomic coordinates of the mouse (test set). We calculated the number of haplotype blocks defined in the mouse genome segment for every projected window. We calculated the Pearson correlation between rat and mouse haplotype block densities (*n* = 54) to obtain the degree of correlation between datasets. This type of analysis was also performed between rat and human and between mouse and human. For all three combinations of species, we also performed the reciprocal analysis by switching the reference and test sets.

To exclude SNP ascertainment bias as a reason for the observed correlations, we reversed rat genotype data while keeping the original genomic locations of polymorphic positions. No significant correlations were found after genotype reversal. Due to low SNP density in the Wade and Daly [3] mouse set, we have excluded it from this analysis.

### Analysis of LD decay for different functional regions.

Using human HapMap public release 19 phased data, we compared polymorphic loci located on autosomes with less than 1-Mb distance between them and calculated |D′| for each pair. We divided SNP pairs into the following functional categories: (1) both SNPs are located in the same gene; (2) SNPs are located in different genes; (3) SNPs are not located within a gene (intergenic); (4) one of the SNPs is located in a gene while the other is in a 30-kb upstream region; and (5) one SNP is located in a gene while the other is in a 30-kb downstream region. We further divided each category into bins by the distance between SNPs with 10-kb steps. For every bin within each functional category, we calculated the arithmetic average and its confidence intervals using a *t*-test (two-tailed with significance threshold of 0.99, α = 0.01). In the case of arbitrary bins, we have calculated the frequency distribution of |D′| values, separately for each category.

### Analysis of excess of SNPs occurring at syntenic positions in mouse and rat.

The SNP frequency in rat (q_r_) was estimated from resequencing results and is consistent with previously reported results [30]. The expected number of SNPs that would be found under random distribution of SNPs in rat and mouse was calculated as N_m_ · q_r_, where N_m_ is the number of mouse SNPs that reside in syntenic regions sequenced in rat (923).

The chance of finding nine or more SNPs at the syntenic positions was calculated as

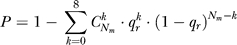



## Supporting Information

Dataset S1 Alignments of SNPs that Occur at Syntenic Positions in Rat and Mouse and Preserve the Same Nucleotide Variants (43 KB PDF)

Figure S1 Haplotype Block Organization in Human Populations (3.6 MB TIF)

Figure S2 Haplotype Block Organization for Different Mouse SNP Datasets (4.1 MB TIF)

Figure S3 LD Decay Plot for Human JPT+CHB (Asian) Population (397 KB TIF)

Figure S4 LD Decay Plot for Human CEU (European) Population (420 KB TIF)

Figure S5 LD Decay Plot for Human YRI (African) Population (419 KB TIF)

### Accession Numbers

Rat SNPs and their genotype information obtained for 41 laboratory rat strains were submitted to the Single Nucleotide Polymorphism Database, dbSNP, (http://www.ncbi.nlm.nih.gov/projects/SNP) and are available under the following accession numbers: ss52089179–ss52090528 and ss52090572.

## Abbreviations

bp: base pairs; kb, kilobases
LD: linkage disequilibrium
Mb: megabases
QTL: quantitative trait locus
SNP: single nucleotide polymorphism
