# Supplementary material for: Haplotype Block Structure Is Conserved across Mammals
Source: PLoS Genet. 2006 Jul 28;2(7):e121. doi: 10.1371/journal.pgen.0020121 (PMC1523234; doi:10.1371/journal.pgen.0020121)
Supplement: Dataset S1 — (43 KB PDF) [file pgen.0020121.sd001.pdf]

Supplementary data 6. Alignments of SNPs that occur at syntenic positions in rat and mouse and preserve the same nucleotide variants

```
Mouse: dbSNP:rs30975573
Rat:   dbSNP:ss52090180

Mouse TCATTCAAAATGGCATA [C/T] AAACACCATGCTAAAGA
      ||||| |||||||||||        || ||||  ||||| |
Rat   TCATTTAAAATGGCATA [C/T] GAATACCACACTAAATA


Mouse: dbSNP:rs30970466
Rat:   dbSNP:ss52090480

Mouse GAAGGGAGGCTTGAACC [C/T] GGGGGCATTGTTGAGAG
      ||||| |||||||||||        ||||||||||| ||||
Rat   GAAGGAAGGCTTGAACC [C/T] AGGGGCATTGTTCAGAG


Mouse: dbSNP:rs30965096
Rat:   dbSNP:ss52089259

Mouse CTTTCTTCCTTCTGCCA [C/T] TCATGCCAACCCTCTTC
      |||||||||||||||         || ||||||| ||||||
Rat   CTTTCTTCCTTCTGCAG [C/T] TCTTGCCAACACTCTTC


Mouse: Sanger:NT_039184.5_5734882  Yalcin et al. 2004:D1Well138:3068637
Rat:   dbSNP:ss52089996

Mouse ATGTTAAGAGGAATATC [A/G] AATGATTTGTGGGAGGA
      ||||      |||||||       | ||| || |  |||||
Rat   ATGT------GAATATG [A/G] AGTGAATTCTCAGAGGA


Mouse: dbSNP:rs31013732
Rat:   dbSNP:ss52090259

Mouse GCTTTATTGGGCTATAT [A/T] TCATGTACTTCCTCATT
      ||||||||||  ||| |       |||| ||||||||||||
Rat   GCTTTATTGGTATAT-T [A/T] TCATTTACTTCCTCATT
```
