# Supplementary figures and images for: Haplotype Block Structure Is Conserved across Mammals
Source: PLoS Genet. 2006 Jul 28;2(7):e121. doi: 10.1371/journal.pgen.0020121 (PMC1523234; doi:10.1371/journal.pgen.0020121)

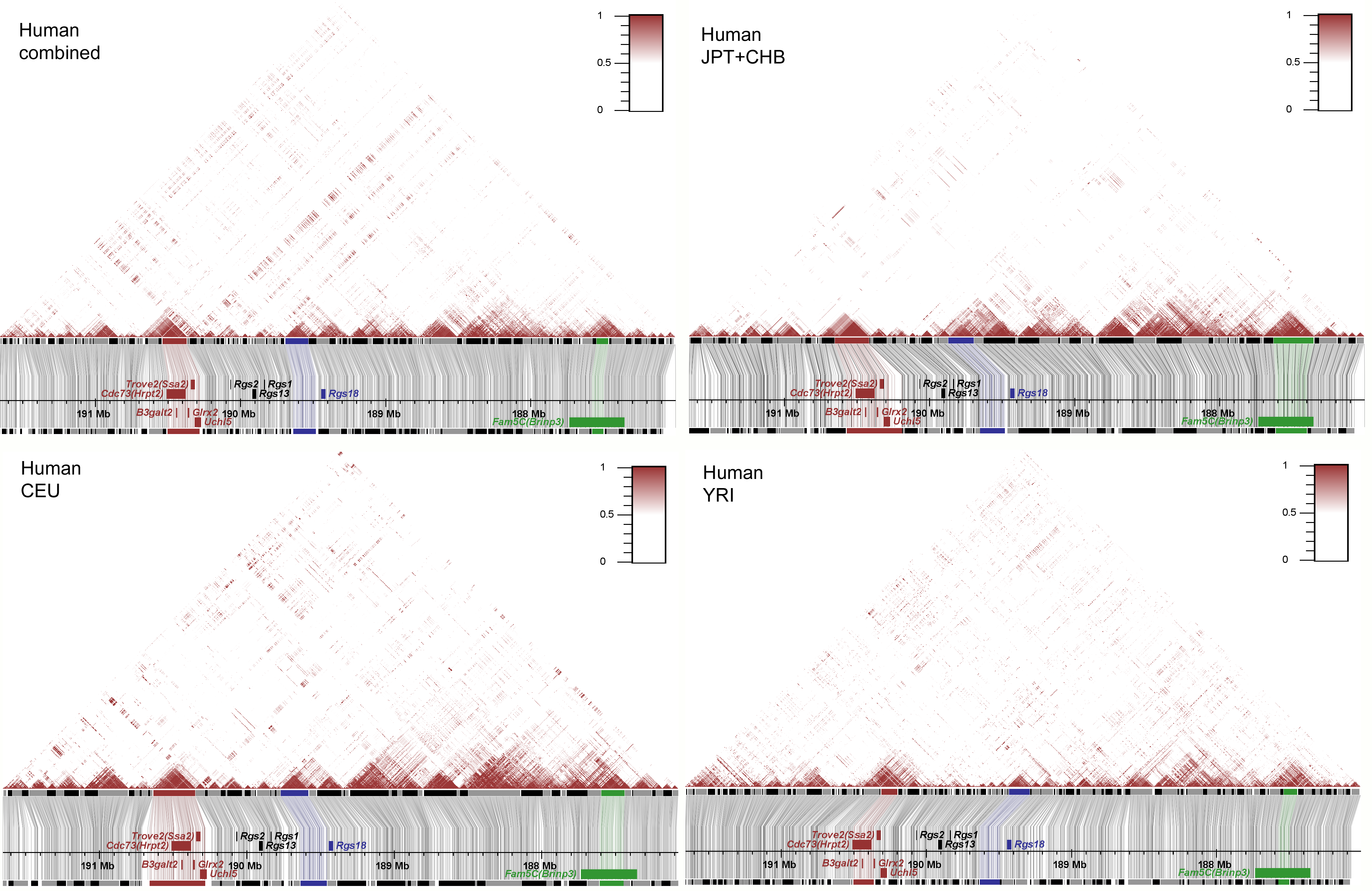

Supplement: Figure S1 — (3.6 MB TIF) [file pgen.0020121.sg001.tif]

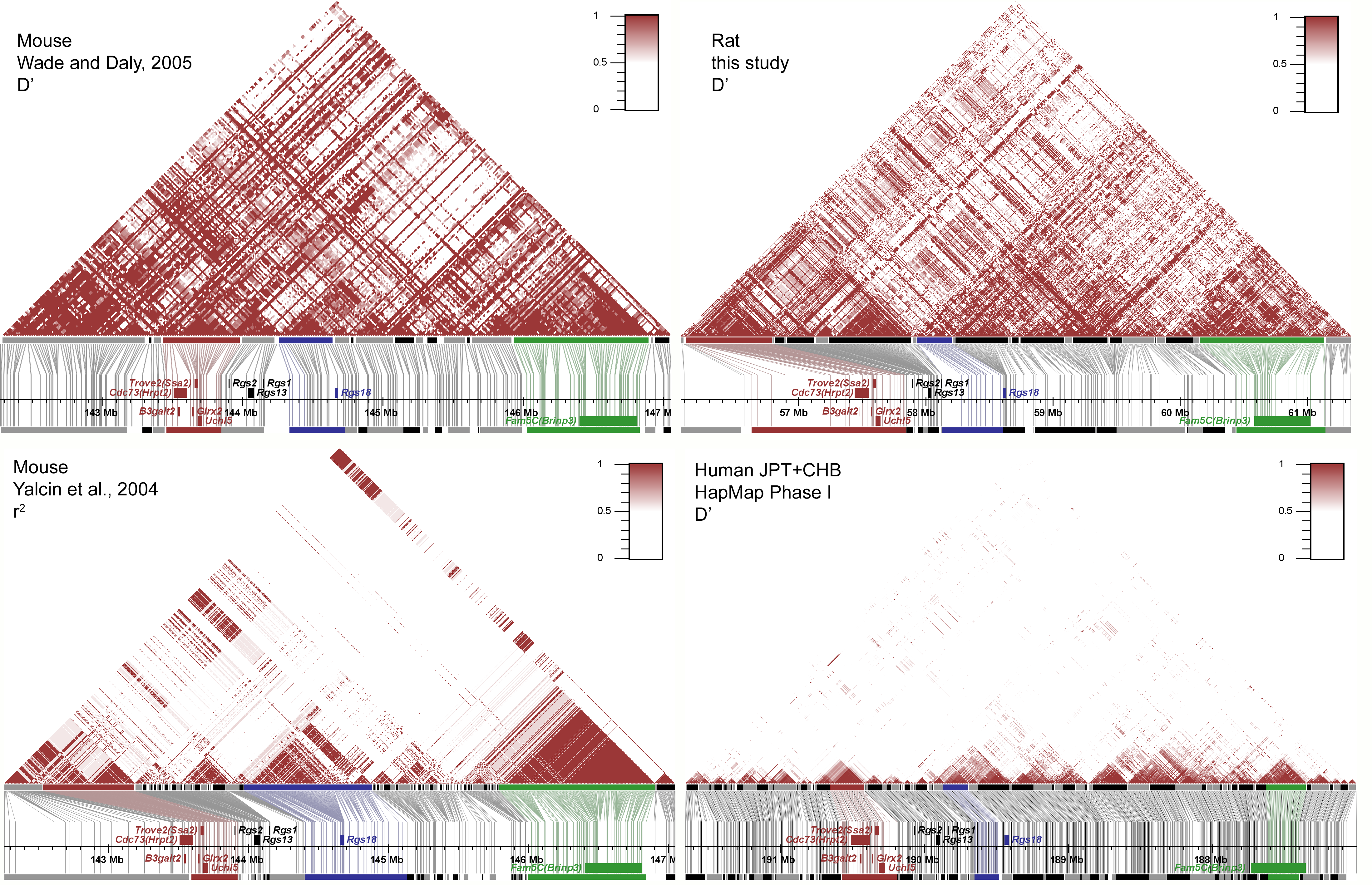

Supplement: Figure S2 — (4.1 MB TIF) [file pgen.0020121.sg002.tif]

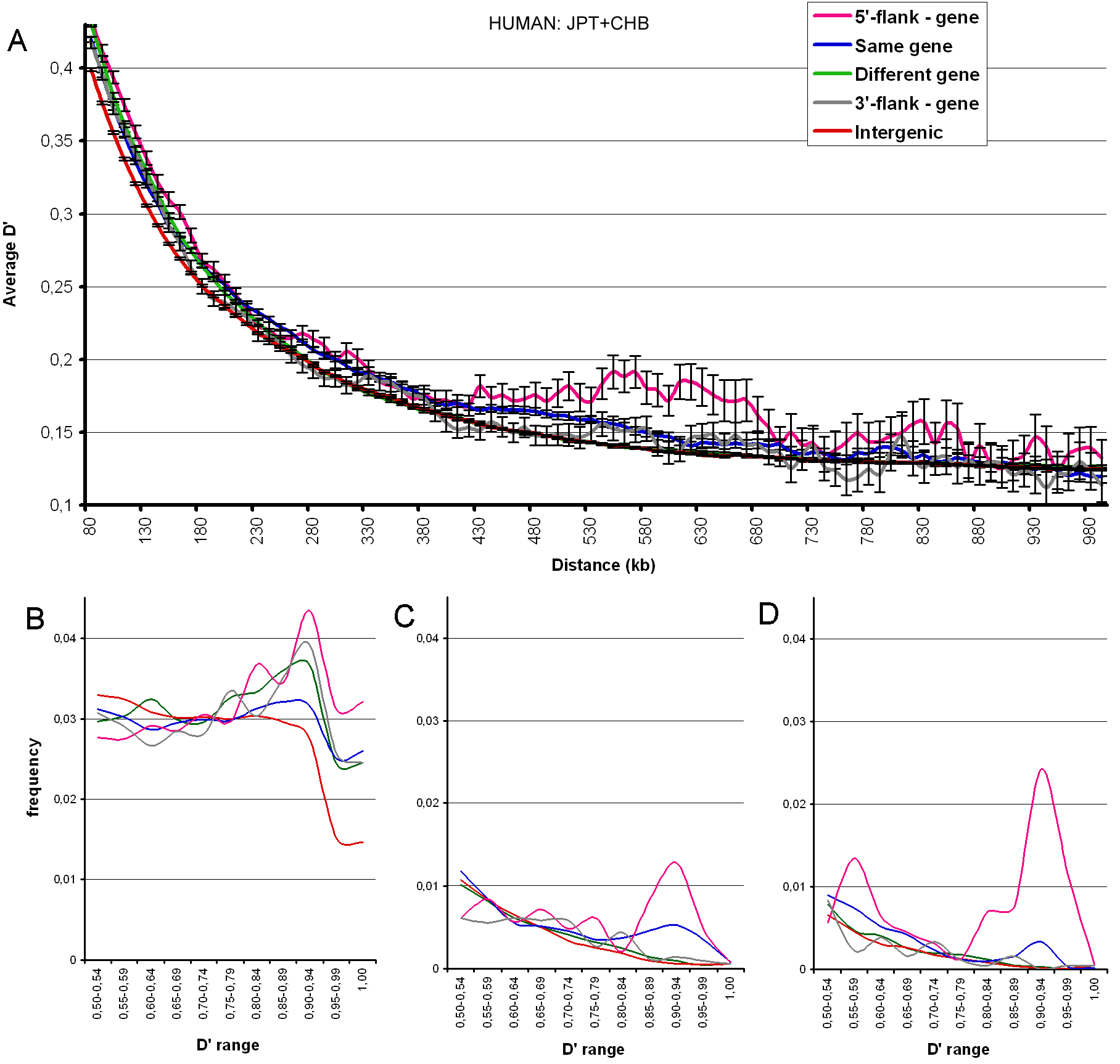

Supplement: Figure S3 — (397 KB TIF) [file pgen.0020121.sg003.tif]

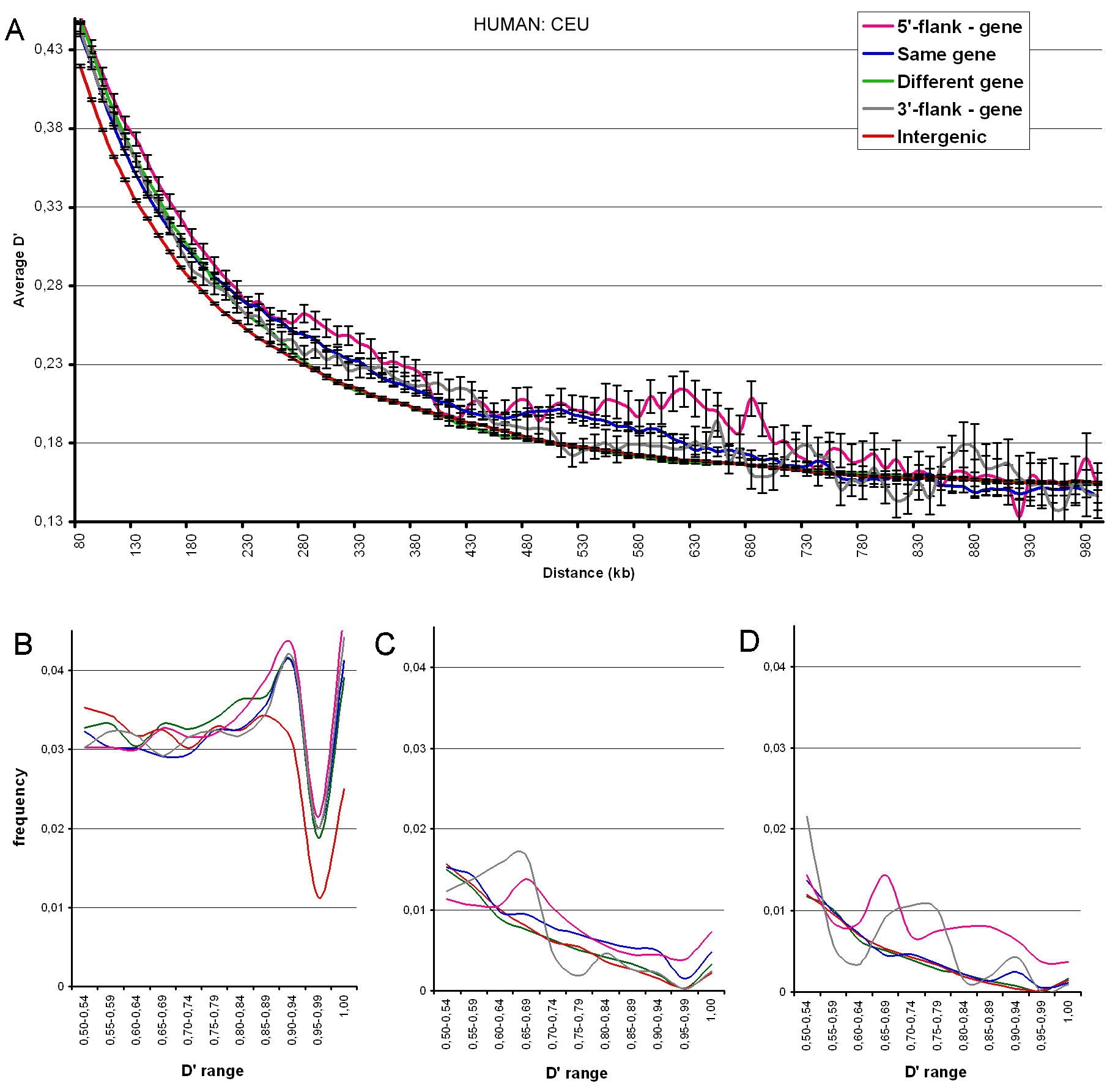

Supplement: Figure S4 — (420 KB TIF) [file pgen.0020121.sg004.tif]

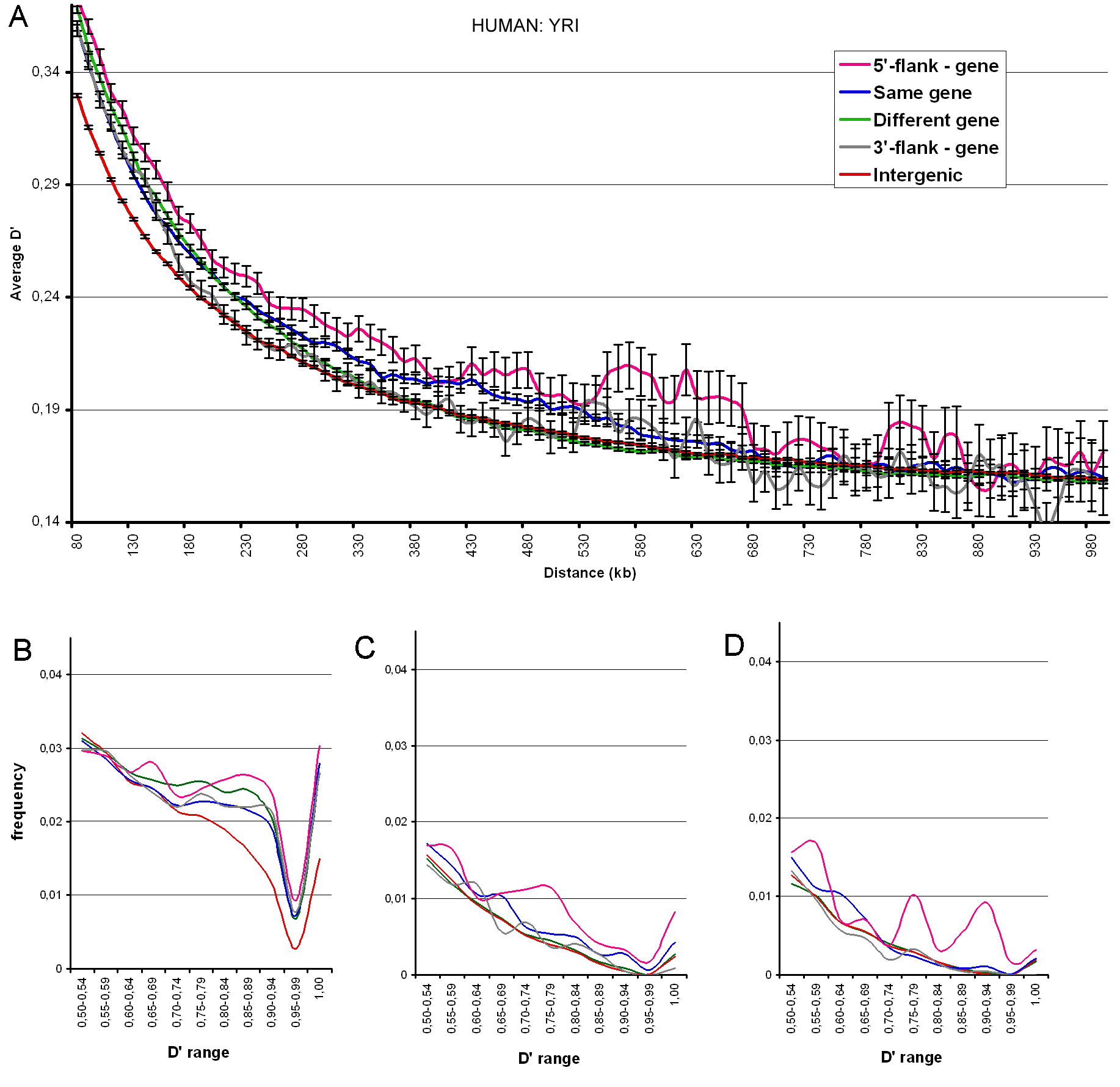

Supplement: Figure S5 — (419 KB TIF) [file pgen.0020121.sg005.tif]
